# Practical approaches to inclusive recruitment practices in Parkinson's disease research

**DOI:** 10.1177/1877718X261440708

**Published:** 2026-05-07

**Authors:** Lana M Chahine, Naomi Louie, Elizabeth Disbrow, Alexis Marbach, Samantha Augenbraun, Bao-Tran Nguyen, Ashani Johnson-Turbes, Carly Parry, Sabrina Avripas, Shivika Chandra, Marissa Dean, Erin R Foster, Deborah Hall, Vanessa Hinson, Camilla Kilbane, Scott A Norris, Ashley Rawls, Cabell Jonas, Ejaz A Shamim, Lisa Shulman, Julia Staisch, Erin Furr Stimming, Tao Xie, Mackenzie Wilcox, Andrew Ameri, Sarah Breaux, Mahesh Padmanaban, Rainer von Coelln, Andrew Singleton, Cornelis Blauwendraat, Sara Bandres-Ciga, Eda Baykal-Caglar, Caitlin Kelliher, Kayleigh Greenwood, Alyssa O’Grady, J Solle, Catherine M Kopil, Maggie McGuire Kuhl, On Behalf of the Black and African American Connections to Parkinson's Disease (BLAAC PD) and the Global Parkinson's Genetics Program (GP2)

**Affiliations:** 1 Department of Neurology, University of Pittsburgh, Pittsburgh, PA, USA; 2 The Michael J. Fox Foundation for Parkinson's Research, New York, NY, USA; 3 Department of Neurology, Louisiana State University Health Sciences Center Shreveport, Shreveport, LA, USA; 4 NORC at the University of Chicago, Chicago, IL, USA; 5 Department of Neurology, UTHealth – Houston, Houston, TX, USA; 6 Department of Neurology, University of Alabama at Birmingham, Birmingham, AL, USA; 7 Program in Occupational Therapy, Washington University in St. Louis, St. Louis, MO, USA; 8 Department of Neurology, Rush University Medical Center, Chicago, IL, USA; 9 Department of Neurology, Medical University of South Carolina, Charleston, SC, USA; 10 Neurological Institute, University Hospitals Cleveland, Cleveland, OH, USA; 11 Department of Neurology, Case Western Reserve University, Cleveland, OH, USA; 12 Department of Neurology, Washington University in St. Louis, St. Louis, MO, USA; 13 Department of Neurology, University of Florida, Gainesville, FL, USA; 14 Department of Neurology, Mid-Atlantic Permanente Research Institute, Kaiser Permanente Mid-Atlantic States, Largo, MD, USA; 15 Department of Neurology, University of Maryland, Baltimore, MD, USA; 16 Department of Neurology, Ochsner Clinic Foundation, New Orleans, LA, USA; 17 Department of Neurology, University of Chicago, Chicago, IL, USA; 18 Global Parkinson's Genetics Program, Chevy Chase, MD, USA; 19 Coalition for Aligning Science, Chevy Chase, MD, USA; 20 Center for Alzheimer's Disease and Related Dementias, National Institute on Aging, National Institutes of Health, Bethesda, MD, USA

**Keywords:** parkinson's disease, genetics, racial health disparities, inclusive research, cohort diversity

## Abstract

In Parkinson's disease (PD), inclusive research recruitment practices are essential to ensure that study findings are generalizable to diverse populations. The definition and implementation of inclusive recruitment practices are guided by the principles of equity, justice, engagement, and sustainability. However, practical implementation guidance is lacking. This paper shares insights from the Black and African American Connections to Parkinson's Disease (BLAAC PD) study, a PD genetics research study being conducted in the United States that enrolls individuals with and without PD. The inclusive recruitment strategy in BLAAC PD centers around four areas: training and working with study personnel toward equitable research practices, partnering with community members, creating culturally resonant study materials, and customizing practices at the local level. We provide practical examples implemented by BLAAC PD to address each of these areas. We share the materials and tools that the study utilizes for site training, recruitment, and community outreach and engagement. These approaches have potential for application in other PD research studies, to achieve greater diversity in PD research.

## Background

The underrepresentation of ancestral, racial, and ethnic groups in Parkinson's disease (PD) research creates an obstacle to a comprehensive genetic, biological, and clinical understanding of the disease^1,2^ and limits the generalizability of results.^3^ Inclusive approaches to engaging and recruiting people who are underrepresented in PD research can address gaps in clinical research and ultimately improve health outcomes for all.^4,5^

Increased awareness of health disparities and the need to advance health equity have prompted the call for inclusive, equitable, and culturally responsive approaches to research.^6^ For example, in 2024 the U.S. Food and Drug Administration published draft guidance encouraging study sponsors to develop Diversity Action Plans for enrolling underrepresented populations in clinical studies.^7^

Inclusive approaches are defined as those that promote the presence of differences such as race, gender, religion, sexual orientation, ethnicity, nationality, socioeconomic status, language, (dis)ability, age, religious commitment, or political perspective.^6^ Inclusive approaches to engaging and enrolling participants in research studies are clearly needed to advance medical science, foster health equity, and rebuild trust with historically marginalized communities. Several groups have put forth data, guidelines, recommendations, and strategies to increase diversity and equity in clinical research.^8–13^ Emerging data from research in PD and other neurodegenerative diseases may represent preliminary signs of success.^9,14,15^ However, a lack of diversity in PD research studies remains a major problem.^16^ Practical guidance on how to accomplish inclusive recruitment practices that lead to increased diversity in PD clinical research studies could thus be of value.

Parkinson's genetics research represents an important example of the critical need for inclusive recruitment practices.^17,18^ Since the initial discovery of genes that contribute to PD in 1997,^15^ much progress has been made in our understanding of the genetic contributions to PD. However, until recently, the majority of PD genetics studies lacked racial and ethnic diversity, and much of what was known about PD genetics came from study samples of predominantly European ancestry.^19,20^ To address critical knowledge gaps around the genetic architecture of PD, the Aligning Science Across Parkinson's (ASAP) initiative^21^ launched the Global Parkinson's Genetics Program (GP2).^22^ GP2 and its contributors aim to collect data and genetic information from over 250,000 individuals worldwide.^22,23^

To increase enrollment of people who identify as Black or African American in GP2 in the United States, GP2 initiated the Black and African American Connections to Parkinson's disease (BLAAC PD)^24^ study in 2021.^25,26^ Participants with and without PD are enrolled in BLAAC PD at twelve sites in the United States for a one-time visit that includes clinical assessments and blood or saliva collection for genotyping. De-identified data are shared with GP2 for integration into the program's open-access dataset.^27^ The BLAAC PD study is ongoing and, as of August 2025, has enrolled over 350 individuals with PD and over 500 individuals without PD who identify as Black or African American.

The strategy for inclusive recruitment practices and community engagement for BLAAC PD centers around four areas: training and working with study personnel toward equitable research practices, partnering with community members, creating culturally resonant study materials, and customizing practices at the local level. Herein, we describe the experience of the BLAAC PD study, sharing practical examples of actions taken to institute inclusive recruitment practices in engaging with individuals and communities for PD research.

## Methods

Since 2022, ASAP's implementation partner, The Michael J. Fox Foundation for Parkinson's Research (MJFF), has collaborated with NORC at the University of Chicago to provide sites with training and technical assistance to promote the engagement and recruitment of people who identify as Black or African American.

The strategy for inclusive recruitment practices and recommendations detailed here stems from a variety of sources including: (1) expertise and experience of MJFF in engaging with the PD community, (2) NORC staff expertise in community-engaged, inclusive, equitable and culturally responsive research, (3) a literature review of the most fundamental aspects of engaging and successfully recruiting people from underrepresented populations for medical/clinical studies, (4) continuing inputs from study teams and subject matter experts, and (5) recommendations from a six-member BLAAC PD Advisory Board convened in 2022 by MJFF to provide strategic guidance and generate recommendations for expansion of the BLAAC PD program. Three of the six Advisory Board members are part of the Black and African American community and the Advisory Board's collective expertise included lived experience as family members of people with PD and as study participants. Other members of the advisory board had experience and/or expertise in clinical care of individuals with PD, scientific expertise in health equity, community-based research, health communication, and translational epidemiology.

### Inclusive recruitment practices: Principles and practical examples

The definition and implementation of inclusive recruitment practices in BLAAC PD were guided by four principles: equity, justice, engagement, and sustainability. Practical approaches and recommendations to realize each principle informed the strategy utilized in BLAAC PD. The four principles are described below, along with recommendations for: (1) training and working with study personnel toward equitable research practices, (2) partnering with community members to strengthen study design and outreach, (3) creating clear, compelling, culturally resonant study materials, and (4) exploring and customizing local recruitment methods. Examples from the BLAAC PD experience are provided, where applicable.

Inclusive recruitment practices have been a top priority for GP2 and BLAAC PD. Site selection, study teams, and the study protocol were all evaluated or created with these principles in mind. The study protocol was designed to prioritize answering key scientific questions while minimizing participant burden. As described elsewhere,^25^ the BLAAC PD study protocol includes collection of a DNA sample and basic information on demographics, PD duration and characteristics, family history, the Clinical Impressions Severity Index for Parkinson's Disease (CISI-PD), olfactory testing, and levodopa intake. Subsequent protocol amendments have added cognitive testing, other non-motor assessments, and environmental/lifestyle measures.

BLAAC PD ensures engagement with and input from community members in an ongoing and iterative process. Feedback from and communication with the BLAAC PD Advisory Board and other community members were obtained during the initial study setup and over time when study amendments were instituted. Anecdotal feedback from potential and existing BLAAC PD participants also informs study modifications.

The approach to inclusive recruitment practices in BLAAC PD has been dynamic and is evolving as the study continues. MJFF, NORC, and site teams meet quarterly. Guest speakers such as genetic counselors and recruitment specialists are invited to these meetings. In a semi-structured format, sites and experts share experiences. Established best practices toward the study's enrollment goals are integrated when applicable and implemented study-wide. Practical examples of how these principles were applied in BLAAC PD are provided below and/or in Tables 1–4 and Supplementary Materials.

**Table 1.** How to train and work with study personnel to institute inclusive recruitment practices: approaches, recommendations, and examples.

Approach	Recommendations	Example applications to BLAAC PD
Build a knowledgeable and empathetic study team	Ensure that the study team has knowledge around past and present research and health care injustices. Pay special focus to potential areas of apprehension such as consent, approved use of samples, etc. Affirm that the study team has knowledge, or share such, around health literacy and socioeconomic diversity and the impacts on access to care and research. Encourage the study team to be mindful of the ways bias could influence the recruitment process and to reflect on biases when talking with potential participants. Be mindful of how medical racism has resulted in reticence in communities of color to engage with research and medical institutions. Use approved recruitment materials and scripts that actively acknowledge the history of structural and medical racism and research exploitation. Engage representative study team members if possible.	Site staff trained on examples of healthcare injustices, atrocities in medical research, and medical racism, such as the Tuskegee experiments BLAAC PD selected study sites that serve diverse populations Study encouraged sites to form study teams that have or strive toward diversity
Prepare staff to interact with potential and enrolled participants	Provide staff with plain language, culturally sensitive talking points, and materials to describe the study and its impacts on PD. Also include talking points to answer anticipated questions, especially around issues such as return of results, use of data/samples, and privacy protections. Keep staff informed on recruitment plans so staff can anticipate and prepare for outreach from potential participants.	Culturally sensitive material and talking points developed (Figure 1) Coordinators provided with talking points regarding how to inform about data/privacy risk and steps taken to maximize confidentiality/privacy The study database was designed to minimize collection of protected health information (PHI). Sites emphasized to participants that any data shared with researchers is anonymized and cannot be linked back to the participant. Data shared with GP2 is all de-identified ICF contains details regarding all entities that have access to the data When partnering with other neurologists or healthcare providers to recruit participants, BLAAC PD encourages sites to ensure those providers are comfortable discussing underrepresentation of minorities in PD research. Sites provide education to referring providers either through grand rounds, brief seminars, or written talking point suggestions.
Provide meaningful support to participants	In addition to compensation for the study visit, consider travel and meal costs for the participant and study companion if helpful to navigate travel or study procedures. Coordinate travel for participants versus asking them to arrange/cover initial costs to alleviate planning burden and upfront costs. Consider other meaningful supports for participants available at your site. For example, offer referral to care navigation services or social worker connections. Consider returning some personal information from the study, such as genetic results and screening outcomes. Plan to (and share with participants how you will) report back overall study outcomes to participants. Note this may require consent to recontact.	BLAAC PD reimburses reasonable travel costs Participants in BLAAC PD are encouraged to co-enroll in PD GENEration^35^, a PD genetics study that provides participants with genetic counseling and results Sites obtain (optional) consent for recontact and distribute newsletters to update study participants who have provided such consent (Supplement pages 27-31)

**Table 2.** How to partner with community members to strengthen study design and outreach: approaches, recommendations, and examples.

Approach	Recommendations	Example application in BLAAC PD
Seek input from partner communities early and throughout study planning	Consult key stakeholders (e.g., potential participants, local community partners) when planning study design (e.g., length of appointment, remuneration and other benefits, recruitment messaging and tactics) Recognizing the plurality of communities, engage a variety of partners Report back on suggestions accepted or declined and share reasons for declination Return to partners when planning amendments or other study changes	At study initiation, a study advisory board was convened The advisory board provided iterative feedback that influenced the content of the protocol and consent form
Use a consensus- based, decision- making framework	Collaborate to identify, articulate, and enhance the value of the study to the community (i.e., identify why a given community would be compelled to join or promote the study). If possible, offer the community group some value for their partnership in this project. You may offer compensation for focus groups or materials review, or direct funds to co-host or leverage an event. Mutually agree upon important issues: how and how often community partners will be involved in the study (e.g., outreach efforts, facilitating referrals, serving as a sample collection site), the importance of proper handling, storage, and confidentiality of samples, consent materials, or other aspects of the study that may involve community sites.	Feedback from the advisory board or community members was sought across several aspects of study design and conduct of the study over time, including but not limited to protocol development, protocol modification, and recruitment materials
Employ a bi- directional learning approach	Initial meetings should be focused on getting to know each other and listening to local needs and capabilities. Do not lead with your study-related ask. Community-based organizations will need training and instruction on the study. Frame the information you share around why their constituents would benefit and how are working with partners like them for a more informed/accessible/productive study. Leverage partners to help site teams understand and reach their partners. Invite partners for information sessions for the study team or have a networking event.	Site teams who meet with community organizations or who attend health fairs (see below) learn from community partners while also offering support and education to partners and their constituents about PD
Engage the community without expectation	Demonstrate an attitude of helping, regardless of whether individuals choose to participate in the study. Create a compilation of resources that study participants might find helpful and/or have requested (FAQs, social service supports and referrals, etc.).	Education materials about PD are provided to sites to share with anyone interested ^36^ Some study site research teams engage in volunteer activities in their communities that are unrelated to the research (e.g., tornado relief, food bank, holiday gift distribution) as a way to be present in, show up for, and give back to the community.
Think and plan “post- study”	Include post-study efforts in the collaboration timeline to demonstrate intentions to remain invested. Reflect on the effectiveness and mutual benefit of activities and determine changes that may be needed to continue or expand the partnership. Create a mechanism for sustained communication. This could include monthly or quarterly check-ins (formal or informal). Celebrate accomplishments and successes.	Permission to contact participants following completion of study activities is obtained at time of consent. Those who provide permission receive updates on the study in the form of periodic newsletters

**Table 3.** Creating clear, compelling, and culturally resonant study materials: approaches, recommendations, and examples.

Approach	Recommendations	Example applications to BLAAC PD
Clearly explain the study, its goal, and why it matters	Include details on why you are doing this research and what it could mean for patients and their families. Articulate why the rationale for inclusion criteria. Emphasize the value of enrolling diverse study populations Include details on how the study is reviewed (e.g., IRB) and how data and samples will be used, stored, and shared. Develop clear and culturally sensitive/compelling outreach materials. Ensure materials are appropriate for a lay audience (8th-grade reading level or below). Use second-person (“you”) versus third-person (“the patient/participant”) when possible.	BLAAC PD informed consent form was designed to address these key points Feedback from community members was elicited on the consent form, protocol, and recruitment materials and incorporated into revisions Examples of culturally sensitive outreach materials are shown in Figure 1 and in the Supplement
Clearly explain the study benefits	Provide information on study remuneration (after approval by appropriate IRB) Incorporate altruistic messaging, noting how participation could help the next generation of patients have access to better care and/or more treatments.	Potential future benefits of research participation to individuals with and without PD were emphasized in the ICF and recruitment materials Participant remuneration was clearly displayed on recruitment materials and in the ICF Efforts were made to ensure participant remuneration in BLAAC PD was commensurate with the schedule of activities and participant time and burden. Therefore, as the protocol was amended to add additional assessments (such as the addition of cognitive testing), participant remuneration was further increased Simple and streamlined approaches to participant remuneration were encouraged where possible, especially those that do not require collection of the participant's personal information (social security numbers, etc.)
Engage participants in the informed consent process	Provide hard copies of the consent form to potential participants so they can read the form as it is explained to them verbally. Know the consent form. Being able to speak to the consent form without appearing to read from a script can build trust and facilitate discussion. Highlight key sections of the consent form that address common questions or concerns about the study. Frequently pause and ask the participant if they have questions or want you to clarify anything.	The consent form was reviewed by community members and revised according to their input Site teams, especially study coordinators, are trained and supported to optimize the informed consent process Asking questions such as “Is this clear?” and “Do you have any questions?” may be more inviting than asking “Do you understand?”: “Is this clear?” puts the onus for any lack of clarity on the study team rather than the investigator; “Do you understand?” may relay a deficiency on the part of the participant.
Empower participants to exercise their agency	Underscore the voluntary nature of participants’ involvement throughout the course of the study. Clearly state the terms of study withdrawal (e.g., previously collected data will not be deleted). Clearly include instructions for withdrawal.	The voluntary nature of participation was clearly indicated in the consent form A clear process for participants to withdraw consent is in place for the study
Leverage partner voices to share study impact and experience	Use testimonials (e.g., quotes, profiles) from representative study participants in promotional materials. Include photos where possible. Allow potential participants to hear the motivations and considerations for joining your research study from those in their community.	Interested BLAAC PD participant advocates and study representatives are engaged where available to promote the study and provide testimonials

**Table 4.** Approaches, recommendations, and examples for exploring local recruitment methods.

Approach	Recommendations	Example applications to BLAAC PD
Meet study participants and potential participants where they are	Strategize outreach to potential study participants not only where they receive care but also where they socialize and access trusted information in a culturally specific manner. Explore site capacity to approach smaller institutions, community centers, and community-serving organizations. The community partners you work with on study input can also help recruit. Explore if your site has a community outreach office with some partnerships already in place that you could leverage for recruitment. Ask your participants where else you should promote the study, and if they could spread the word in their networks. Share study recruitment materials with them. Track outreach efforts and contacts made to evaluate and iterate your recruitment strategy.	Encouraged sites to engage with local community directly or through institutional community outreach resources Partner with community-based health care organizations (federal, state, city, or non-profit organization-run, depending on location) Identified community ambassadors and asked them to distribute recruitment materials within their networks Asked local PD support groups to distribute recruitment materials to their distribution lists (examples from the BLAAC PD partner toolkit are in supplement pages 32-37)
Using media to support study recruitment	Ask your site's public relations or marketing teams if they can offer any support. Consider owned media. This is an audience that you already have. Perhaps it's an email list, table tents in a cafeteria, or screensavers in waiting rooms. Consider earned media. This is the media that you pitch for. Send a news alert to local radio or television about the study. Think about the “hook.” Is it an awareness month tied to the disease, or was there a recent discovery you are building upon? Do you have a great personal story from a participant? Consider paid media. This is social media, online or print advertisements that you pay for. This often requires only some light design work but can be expensive. Adapt the communication channel to the audience. Eligible older adults may respond to print media, but social media may reach younger caregivers of eligible participants. Try multiple channels and messages. Consider the voices in your campaigns. Do you have study doctors and participants who can share their stories on the study's impact and goals? Complete mock interviews to prepare. Consider how you want people to contact you. Make it easy for interested participants to take action. Include a phone number or a link to a website. Try a QR code for print items, but include a website too, in case people don’t know how to use a QR code.	Radio ads aired on radio stations that broadcast near study sites Newspaper ads have been printed in local newspapers Information on BLAAC PD included in site's institutional newsletters Flyers or brochures about the study distributed throughout campuses at sites affiliated with universities (Supplementary material pages 10-19) A centralized phone line was created to reduce time and effort on screening potential participants at the site level. This also improves participant experience through streamlining and consistency with a more direct way to engage and get information about the study. MJFF advice/approach to media is shown in the supplement (pages 2-9)

#### Equity: Eliminating inequities in knowledge & treatment

Equity in access to and delivery of high-quality healthcare is a critical prerequisite for equity in clinical research. Health inequities in PD are well documented, especially racial, ethnic, and geographic (rural vs. urban) disparities in PD diagnosis and treatment.^28^ While addressing these inequities requires systemic changes across multiple areas beyond the scope of BLAAC PD as a study, they are a key priority within the clinical care delivery systems at study sites.

As with any research study, ensuring that participants are aware of and maintain their agency while participating is essential.^29^ One way to encourage and maintain participant empowerment is to be open and transparent about the nature of research being conducted. This includes proactively sharing information such as why the study is being conducted, what participants will be asked to do in the study, and how their samples will be secured and used.^30,31^ Awareness of health literacy challenges is important.^32^ It is recommended that patient-facing health information not exceed an eight-grade reading level in the United States,^33^ though in some settings, lower reading levels may be more appropriate. This is particularly relevant when study-related goals or assessments that are going to be discussed with potential participants relate to complex concepts such as genetics. Active feedback from community partners is invaluable as messaging around the study and its value proposition are conceptualized and developed. Recognizing the diversity of communities (across and among demographic groups) requires tailoring materials and approaches to reflect that diversity, which will increase trust and willingness to participate.^34^ In turn, this will improve the breadth of scientific knowledge on PD across racial, ethnic, and ancestral groups.

In BLAAC PD, ensuring that participants were aware of and understood various aspects of the study and the meaning and implications of their participation was achieved in several ways. As in most studies, the informed consent form (ICF) content was of particular priority. Site team members were trained on the informed consent process (Table 1).^35,36^ In addition, the study partnered with community members to ensure the ICF and other study materials were informative and understandable (Table 2). Specifically, Black or African American individuals with PD and/or their family members provided feedback on the ICF, protocol revisions, and recruitment materials, and their feedback was incorporated. For example, when the study modified one of its case report forms to collect more detailed information on family history, community members reviewed a draft of the form. Their feedback highlighted the need to more clearly explain the difference between a full sibling and a half sibling, and the family history case report form was revised to incorporate this information.

**Figure 1.**
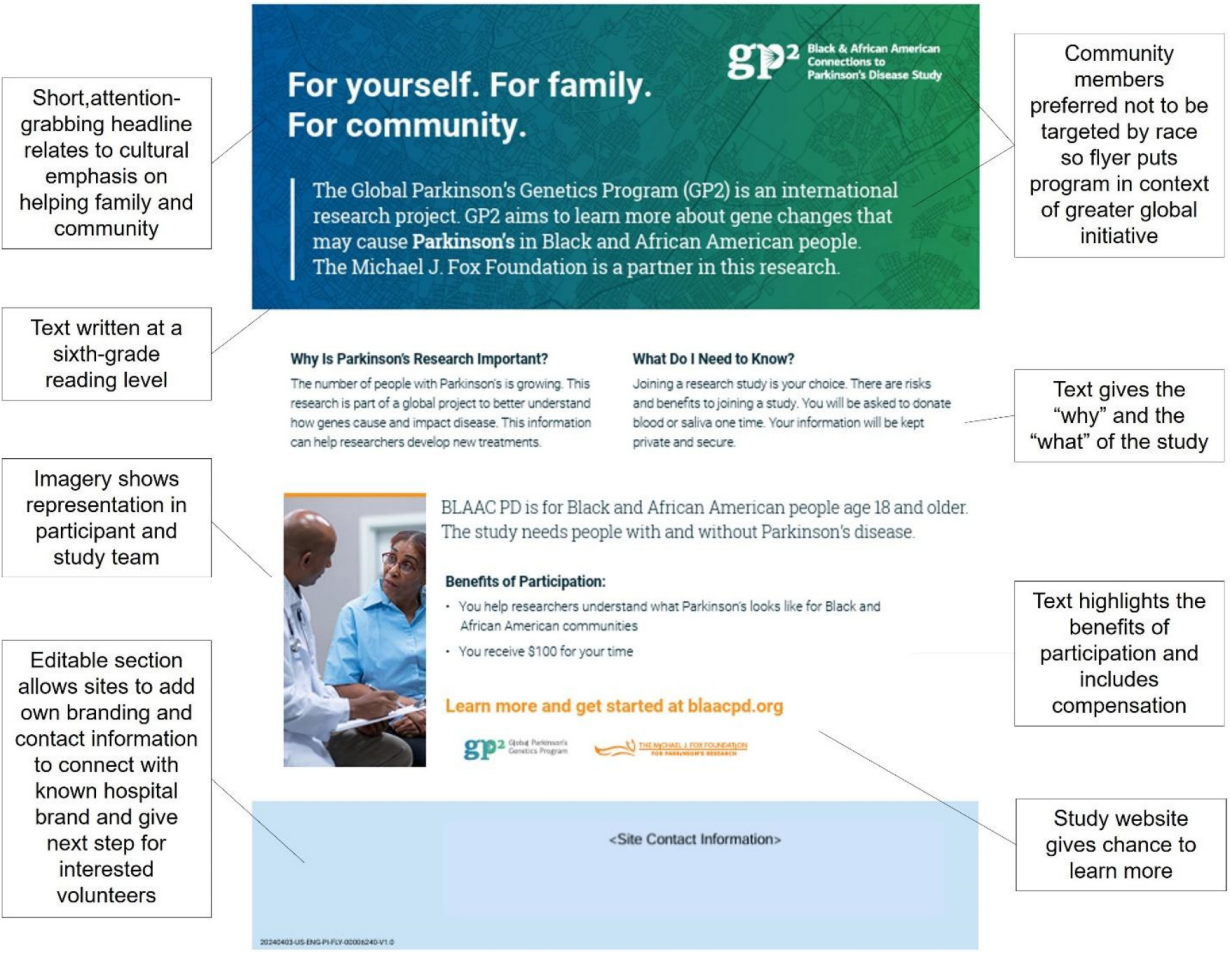
Annotated example of BLAAC PD flyer explaining inclusive design considerations.

Furthermore, several participant-facing materials in different formats were created and distributed to sites to help inform and educate potential participants (Table 3 and Supplementary Material). Community feedback was solicited in the development of such materials, and materials were revised accordingly. Images were intentionally inclusive and representative. Wording on study materials was simple, with a clear message, while also being informative. An annotated example of a study flyer is shown in Figure 1, and other examples of materials developed for BLAAC PD are provided in the supplement including flyers and brochures (supplement pages 10–19), frequently asked questions (FAQs) (supplement pages 20–26), participant newsletter (supplement pages 27–31), and partner toolkit (supplement pages 32–37), including social media outreach examples (supplement page 37). Study teams, and especially study coordinators, received talking points and ongoing training and support to provide them with the knowledge, communication skills, and confidence to speak about complex topics such as PD genetics and data privacy. At regularly scheduled meetings that brought teams together within and across sites, site teams shared their experiences, including which approaches seemed more successful than others, and where additional training or support was needed.

Finally, it is crucial to acknowledge that we all carry preconceived and often subconscious biases and stereotypes. Maintaining a self-reflective approach and checking one's biases throughout the recruitment and engagement process can help to build mutual trust with individuals eligible for the study.^34^ In BLAAC PD, unconscious bias and other related topics were addressed in a webinar presented to site teams (see below).

#### Justice: Address past and present injustices

Cases of historical medical exploitation are well-documented (e.g., the Tuskegee study, Henrietta Lacks, and the Havasupai tribe).^37,38^ As such, it is important to be mindful of attitudes that minoritized communities may hold toward medical research. It is crucial to recognize that these attitudes are based on both historical and contemporary experiences. Even if current community members were not alive during the era of segregated care delivery and abuses of people of color in the context of science, communities recall medical and institutional racism. Moreover, this legacy is augmented by ongoing experiences of discriminatory practices that contemporary individuals face when interacting with the medical community.^39,40^

One reported best practice for addressing mistrust toward the medical profession is for health care providers and study teams to acknowledge and denounce well-known cases of historical medical exploitation.^29,41^ This recommendation was echoed by BLAAC PD Advisory Board members, who stressed the importance of acknowledging historical racism in communication with participants. Additionally, the study FAQ pointedly discusses the allowed uses for all contributed study data and samples (Supplementary Material).

#### Engagement & sustainability: Engage local communities in reciprocal relationships and sustain relationships beyond the scope of the study

##### Engagement with Community Members

For successful engagement of local communities—the communities from which the study is recruiting participants—resources and training for skills development or capacity building for the study teams are a core component of improving diversity in research cohorts (Table 4).^42,43^ Co-learning forums where sites share their own wins and challenges are helpful engagement and learning tools. Training sites in best practices for communication is a critical component.^44^ The training session may benefit from discussing and modeling communication skills to engage potential study participants in clinic and community settings. A brief didactic presentation on communication skills and decision-making processes in recruitment, followed by group role-play exercises practicing common recruitment scenarios, is one possible format. Topics may include verbal and non-verbal communication approaches to build rapport and trust, potential study participants’ decision-making processes and questions, and communication strategies to support decisions to participate or not in the study. Webinars with ample opportunity for questions and discussion may be a useful format to deliver key information to study sites.

In BLAAC PD, a range of topics were covered in BLAAC PD forums and training, including basics of PD genetics, forms of media, how to best utilize health fairs for recruitment, development of various communication skills, and communication around complex topics. To improve communication about genetics with participants, webinars covered basics of how to explain genetics and genetics research, basics of genetic counseling, and importance of genetics research to understanding of PD. Another session covered specific and intentional strategies to engage the Black and African American community in research, featuring a PD researcher working externally to BLAAC PD. Opportunities for coordinators to share their lived experiences and practical strategies and/or workflow changes to support BLAAC PD recruitment also fostered co-learning and collaboration.

##### Engagement with Community-Based Organizations

Literature underscores the need to engage and develop local reciprocal relationships with community-based organizations in which a mutual exchange of support occurs between both partners.^40,41^ Study sites could seek to establish connections with local community-based organizations (CBOs), whether organizations focused on PD or not, emphasizing how study involvement could help CBOs further their goals of community support. It is important that sites consider what other value they could offer, such as disease education and facilitation of clinical care.

When forming relationships with CBOs, collaborative decision-making can build trusting relationships, engendering knowledge co-production and exchange for relevant, actionable evidence that best serves communities.^45,46^ Understanding the value proposition of the study from the perspective of the CBOs/communities requires sites to learn from the CBOs about their perspectives and needs, as well as concerns of community leaders/members.^8,34^ One recommended approach toward establishing collaborative engagement between a site and CBO/community is to form dyads or breakout groups for open, reciprocal discussions that pair CBO leaders/community members and site staff members, and have the pairs report on a particular decision to the larger group.^47,48^

There are several barriers that may impede CBO engagement with research studies. Barriers include poor study infrastructure, lack of staff continuity, and/or no sustainability plan.^42^ CBOs may be hesitant to engage if they expect a partnership to be temporary and predicated on their involvement with a particular project (i.e., the study). Acknowledging barriers and setting expectations at the outset that collaboration, consistent communication, and joint work with the CBO is intended to be a long-term commitment — and then acting on that commitment — can facilitate successful engagement with local organizations.^49^ Additionally, reporting back on how suggestions were integrated or considered can build trust and support sustainability.

At the local level, BLAAC PD study sites engaged with the community and CBOs in various settings, including health fairs, educational institutions, and community health centers. Health fairs are viable engagement and recruitment methods; however, in the context of PD research, are more likely to result in recruitment of controls rather than PD cases. Table 5 provides tips for planning, attending and following up at health fairs and other community-driven health initiatives as part of engagement and recruitment efforts. Events are best attended by staff with local familiarity and connection to facilitate communication and trust building.^50^ For example, many BLAAC PD study teams are engaged with entities that either organize or attend health fairs. The health fairs may be general community health fairs or those specific to older adults or certain communities, such as the Black or African American community. Study teams attended these health fairs, setting up booths to provide attendees with information about PD, PD research, and BLAAC PD. In addition to educational materials around PD and brain health, study teams provided attendees with some “stuff we all get” (SWAG), such as T-shirts and hats with the study logo.

**Table 5.** Strategies and tips for attending community health fairs or other off-site events.

**POSSIBLE VENUES / COMMUNITY PARTNERS**
Local health fairs are especially useful for engagement and recruitment of controls.
Events focusing on healthy aging, brain health, etc., may be of particular importance.
Institutional or foundation-based PD awareness events or fundraisers (especially useful for engagement and recruitment of cases).
Consider partnering with other disease-focused organizations, such as those raising awareness or recruiting for Alzheimer's research.
Utilize online resources and search engines to identify local groups that host events and/or local health fairs.

**LOGISTICAL CONSIDERATIONS**
Define your goals upfront: Will you be attending the event to provide education, advertise the study, and/o or to attempt to recruit and collect study data on-site?
Determine the rules, regulations, resources, and support provided by the community event organizers to achieve your goals.
Obtain your institution's regulatory approval for whatever activities will occur at the fair. Special approvals may be needed to obtain consent and perform study activities on-site.
Try to visit the venue ahead of time to know what to expect and to determine available resources.
Take a group to help staff your table. Assign specific roles: a greeter, someone to assist with handing out supplies, and, where applicable, team members to take charge of specific regulatory or protocol aspects (such as privacy, consent, specimen, data collection, etc.).

**ESTABLISHING AND MAINTAINING RELATIONSHIPS WITH ORGANIZERS AND ATTENDEES**
Have a brief, catchy “elevator pitch” about the study ready. Include the “why” of the study and not just the “what.”
Be prepared to hear “No.” Remain accepting of no as a response to invitations for study participation. Do not let “no” responses discourage or dissuade you from sharing valuable information with all potential participants.
Provide clear and direct contact information for your team to interested individuals.
Provide contact information to potential participants and manage expectations. Communicate who they can expect to hear from and provide a realistic timeframe in which your team will reach out (e.g., “by early next week” or “within 2-3 business days”).
Make your table/booth/stand attractive. Use visible and eye-catching signs and banners for your table, including a tablecloth with the study logo on it.
Bring study or site-branded handouts, like pens or stress balls. Be generous with SWAG where possible.
Use interactive activities to bring attention and engagement (e.g., offer candy, jeopardy/trivia boards, a grip strength test, a spinning wheel to spin to win prizes).
Enrollment of interested participants is more successful when the study team is able to contact potential participants rather than awaiting potential participants to contact the study team. (Permissions from event organizers or your institutional regulatory bodies may be needed to collect contact information of attendees.)
Where possible/feasible, collect attendees’ contact information and permission to contact them after the event.
Collect contact information in a delicate manner. Ask potential participants for their preferred mode of communication and the best time of day to contact them.
To help maintain privacy, avoid a sign-up sheet where possible. Instead, use individual forms that can be stored in a box, or create a web-based form on a tablet or laptop.
Introduce yourself to other exhibitors. Explore the potential to partner with them on recruitment or to attend future events.
After the event, send thank-you letters to venue / hosts / partners where applicable
Collect relevant data to support evaluation. Track the number of people who approached the table, the number of materials distributed, and the number of individuals from whom contact information was received.
Some health fairs collect data on attendee characteristics. Request this from organizers where available / applicable.

**SUPPLIES**
Study literature and marketing materials
Stuff we all get (“SWAG”)
Education materials and resources about PD for non-PD and PD audiences

**ONSITE RECRUITMENT AND STUDY ACTIVITIES: SUPPLIES AND SPECIAL CONSIDERATIONS**
A permit may be required to collect blood samples at health fairs. Please check your institutional, local, and state requirements before collecting blood.
Not all venues are conducive to on-site recruitment and conducting study activities. Study teams should familiarize themselves with the venue and resources available before attempting to conduct on-site study activities.
Participants should sign two consents—one for them to take home, one for the study site.
Separate, locked storage for paperwork containing PHI
Pens and Markers (for consent and sample labelling)
Blank Consent Forms (twice as many as you anticipate needing)
Blank Participant Compensation Forms
Confidential Participant ID list/tracker
Participant Compensation
Privacy screen or space divider
The consent process needs to occur in a “private setting” (separate room or behind a privacy screen or partition)
Store signed consents and compensation forms in a separate container from samples.
Quiet place without distractions to administer certain tests, such as cognitive tests
Quiet place without odors to administer smell test (for example, away from food stands)

**SAMPLE COLLECTION SUPPLIES AND SPECIAL CONSIDERATIONS**
Specimen collection supplies (blood tubes, alcohol wipes, tourniquets, needles, syringes, saliva kits, etc)
Chair and bolsters for specimen collection
Labels (pre-printed/blank for biosamples)
Portable refrigerator for blood (in BLAAC PD blood samples require refrigeration (+2C to +6C) from the time they are drawn until they can be placed in a -80C freezer, which must occur on the same day they are drawn)
Biosamples (e.g., saliva and/or blood) should be stored in a locked container or refrigerator that is supervised by a member of the study team.
Blood and other specimens must be obtained following the study and institution's standard lab protocols/processes.

It is important that the study teams engage with the community and specific CBOs beyond a single health fair. Consistent presence and engagement at health fairs can be meaningful, not only to community members in attendance, but also to community leaders and CBOs who may have booths at these fairs. Thus, the goal for the research team is to develop a bidirectional and mutually beneficial relationship with CBOs. BLAAC PD site teams provide seminars and materials to educate CBO staff or community members about PD^36^ and research in general. Beyond health education and research, some study sites used creative engagement approaches such as holding Bingo events at senior citizen centers.

#### Media campaigns

Common recruitment efforts may also include owned, earned, or paid media campaigns. A sample presentation on this topic, used for the BLAAC PD study, is included in the Supplementary Material. Another common recruitment effort is to use direct mail, shown to be more effective at recruiting historically marginalized populations than mass media.^51^ In the Supplementary Material (pages 10–26), we provide recruitment materials from the BLAAC PD and GP2 program for reference and adaptation.

## Discussion

Drawing upon prior experience, contributions from study sites, and real-time study activities, we have presented a strategy for inclusive research recruitment and community engagement, providing practical examples of implementation and resources utilized in the BLAAC PD study to recruit individuals in the United States with and without PD who identify as Black or African American. The strategy and tools used to implement it may be applied globally to a broad array of PD clinical research studies that seek to engage and recruit ethnically/racially diverse groups for more inclusive disease understanding and treatment development. That said, a key observation is that approaches to recruiting groups underrepresented in research must be adapted^52^; transferability of the specific approaches for inclusive practices we describe will likely require modification depending on the context of use. One ‘size’ or set of offerings does not fit all, and racial/ethnic communities cannot be viewed as a monolith. Within the context of a multi-site study, approaches to culturally responsive engagement and recruitment must be tailored to the local community's unique needs and preferences. Partnering with members from the community, and obtaining input from them and study participants over time, is critical toward understanding the latter. For example, in BLAAC PD, regional differences in preferred terminology were noted, and study materials and messaging were modified accordingly. In addition, adaptation of site team building, training, and, more broadly, design and implementation of study operations requires consideration of the sites’ unique resources, capacities, and challenges. In areas with limited local resources for PD research, partnering with national and international PD organizations and foundations could be useful.

In the context of PD research, a main goal of inclusive recruitment practices is to increase diversity in PD research cohorts. This allows for improved generalizability of research results to the PD population. On the other hand, in PD genetics research in particular, cohort diversity is critical towards informing development and delivery of personalized care. Thus, whether improving our understanding of PD at the population level or informing knowledge of PD at the individual level, the ultimate measure of success of inclusive recruitment practices over the long-term is reduced disparities and, ultimately, equity in delivery of care to all people with PD. However, in the shorter-term, some measures of success may be considered.

Keeping in mind that inclusive recruitment practices, such as team building and community engagement programs, are an ongoing process to establish enduring partnerships rather than short-term transactional relationships, one broad measure of the impact of inclusive recruitment practices is the success of a given study in its recruitment goals. This is exemplified, for example, in BLAAC PD's success to date in recruiting a sample traditionally under-represented in PD research, including recruitment of the majority of controls through efforts related to community engagement.^53^ Further research is needed to understand how to tailor these and other strategies for inclusive recruitment practices for other clinical research settings and goals (e.g., institutions of different sizes, geographic regions, populations of focus). This work could shed light on appropriateness of content within a given context, project roles, necessary adaptations, feasibility, and potential for sustainability. An outcomes-focused evaluation could also determine whether the strategy is practical and actionable (i.e., suitable for everyday use), responsive to the needs of the field, and contributes to inclusive recruitment practices for clinical research. Such an outcomes evaluation could include metrics on uptake (e.g., number of downloads, page views of online study-related information), cost-benefit analyses, and qualitative measures (e.g., focus groups and key informant interviews conducted with different types of clinicians and administrators) to test user experience and identify potential areas for improvement.

The need to improve diversity in PD research cohorts is widely recognized. Here, we present the strategy being implemented in BLAAC PD to support inclusive recruitment, including a compendium of actionable tips and approaches for PD researchers and study teams. We recognize that this and similar strategies will require systematic evaluation to better understand their effectiveness, potential applications, and how key elements may need to be adapted across settings. We encourage PD researchers to explore, apply, and adapt these and other inclusive recruitment practices to advance diversity in Parkinson's disease research.

## Supplemental Material

sj-pptx-1-pkn-10.1177_1877718X261440708 - Supplemental material for Practical approaches to inclusive recruitment practices in Parkinson's disease research Supplemental material, sj-pptx-1-pkn-10.1177_1877718X261440708 for Practical approaches to inclusive recruitment practices in Parkinson's disease research by Lana M Chahine, Naomi Louie, Elizabeth Disbrow, Alexis Marbach, Samantha Augenbraun, Bao-Tran Nguyen, Ashani Johnson-Turbes, Carly Parry, Sabrina Avripas, Shivika Chandra, Marissa Dean, Erin R Foster, Deborah Hall, Vanessa Hinson, Camilla Kilbane, Scott A Norris, Ashley Rawls, Cabell Jonas, Ejaz A Shamim, Lisa Shulman, Julia Staisch, Erin Furr Stimming, Tao Xie, Mackenzie Wilcox, Andrew Ameri, Sarah Breaux, Mahesh Padmanaban, Rainer von Coelln, Andrew Singleton, Cornelis Blauwendraat, Sara Bandres-Ciga, Eda Baykal-Caglar, Caitlin Kelliher, Kayleigh Greenwood, Alyssa O'Grady, J Solle, Catherine M Kopil, Maggie McGuire Kuhl and On Behalf of the Black and African American Connections to Parkinson's Disease (BLAAC PD) and the Global Parkinson's Genetics Program (GP) in Journal of Parkinson's Disease
